# Long non-coding RNA as a potential diagnostic biomarker in head and neck squamous cell carcinoma: A systematic review and meta-analysis

**DOI:** 10.1371/journal.pone.0291921

**Published:** 2023-09-21

**Authors:** Mahdi Masrour, Shaghayegh Khanmohammadi, Parisa Fallahtafti, Nima Rezaei

**Affiliations:** 1 School of Medicine, Tehran University of Medical Sciences, Tehran, Iran; 2 Non-Communicable Diseases Research Center, Endocrinology and Metabolism Population Sciences Institute, Tehran University of Medical Sciences, Tehran, Iran; 3 Research Center for Immunodeficiencies, Pediatrics Center of Excellence, Children’s Medical Center, Tehran University of Medical Sciences, Tehran, Iran; 4 Network of Immunity in Infection, Malignancy and Autoimmunity (NIIMA), Universal Scientific Education and Research Network (USERN), Tehran, Iran; 5 Department of Immunology, School of Medicine, Tehran University of Medical Sciences, Tehran, Iran; University of Rome La Sapienza Sapienza Faculty of Medicine and Dentistry: Universita degli Studi di Roma La Sapienza Facolta di Medicina e Odontoiatria, ITALY

## Abstract

**Background:**

Head and neck squamous cell carcinoma (HNSCC) is a group of malignancies arising from the epithelium of the head and neck. Despite efforts in treatment, results have remained unsatisfactory, and the death rate is high. Early diagnosis of HNSCC has clinical importance due to its high rates of invasion and metastasis. This systematic review and meta-analysis evaluated the diagnostic accuracy of lncRNAs in HNSCC patients.

**Methods:**

PubMed, ISI, SCOPUS, and EMBASE were searched for original publications published till April 2023 using MeSH terms and free keywords “long non-coding RNA” and “head and neck squamous cell carcinoma” and their expansions. The Reitsma bivariate random effect model pooled diagnostic test performance for studies that reported specificity and sensitivity; diagnostic AUC values from all trials were meta-analyzed using the random effects model with the inverse variance method.

**Results:**

The initial database search yielded 3209 articles, and 25 studies met our criteria. The cumulative sensitivity and specificity for lncRNAs in the diagnosis of HNSCC were 0.74 (95%CI: 0.68–0.7 (and 0.79 (95%CI: 0.74–0.83), respectively. The pooled AUC value for all specimen types was found to be 0.83. Using the inverse variance method, 71 individual lncRNAs yielded a pooled AUC of 0.77 (95%CI: 0.74–0.79). Five studies reported on the diagnostic accuracy of the MALAT1 lncRNA with a pooled AUC value of 0.83 (95%CI: 0.73–0.94).

**Conclusions:**

LncRNAs could be used as diagnostic biomarkers for HNSCC, but further investigation is needed to validate clinical efficacy and elucidate mechanisms. High-throughput sequencing and bioinformatics should be used to ascertain expression profiles.

## Introduction

Head and neck squamous cell carcinoma (HNSCC) is a heterogeneous group of malignancies arising from the epithelium of the lip, oral cavity, nose, sinuses, nasopharynx, oropharynx, hypopharynx, and larynx. Based on tumor location, HNSCC is divided into different types, including tongue squamous cell carcinoma (TSCC), oral squamous cell carcinoma (OSCC), laryngeal squamous cell carcinoma (LSCC), and nasopharyngeal carcinoma (NPC) [1].

HNSCC is the sixth most common cancer globally and one of the most common causes of mortality due to cancer, accounting for 450,000 deaths worldwide in 2018 [2]. The incidence of head and neck cancers is predicted to rise to 856,000 cases annually by 2035 [3]. Despite the efforts to treat HNSCC, results have remained unsatisfactory, and the death rate is high [4]. Most cases of HNSCC are not diagnosed until regional lymph node metastases. As poor prognosis and relatively high mortality of HNSCC are primarily due to the high rates of invasion and metastasis [5], early diagnosis of HNSCC has clinical importance.

Histopathological evaluation of the tissue obtained by tumor or neck mass biopsy is currently the gold standard for diagnosing HNSCC [6]. Incisional biopsy, excisional biopsy, and fine needle aspiration (FNA) are used depending on the primary tumor [6]. However, major tissue biopsy drawbacks include false acquisition of samples due to tumor heterogeneity, patients’ discomfort, and difficulty in diagnosing regional metastasis [7]. Recently, there has been a rising interest in liquid biopsies as an alternative non-invasive method for molecular characterization of HNSCC [8]. Due to their minimally invasive nature, liquid biopsies have been known as a novel approach for screening, diagnosing, and monitoring HNSCC [8].

In recent years, there has been a growing emphasis on various categories of non-coding RNAs, such as long non-coding RNAs (lncRNA), microRNAs, and circular RNAs (circRNAs), and their potential diagnostic, prognostic and therapeutic significance in various types of cancer, specially colorectal cancer [9–11], breast cancer [12, 13], endometrial cancer [14, 15], gastric cancer [16, 17], thyroid cancer [18–20], and solid tumors [21–23]. Several studies have highlighted changes in coding and non-coding RNAs in HNSCC patients, which have demonstrated the critical roles of these molecules in the pathogenesis of HNSCC [24, 25]. One of the non-coding RNA molecules involved in the pathogenesis of HNSCC is lncRNA. LncRNAs are a group of non-coding RNA molecules with at least 200 nucleotides [26]. Evidence has shown that lncRNAs play a pivotal role in various cancers, such as gastric, colon, and prostate [27]. Alterations in the expression of lncRNAs can cause changes in cellular proliferation, apoptosis, and invasion [27]. The abnormal expression of lncRNAs from tissue, serum, saliva, or urine samples is detected by using molecular methods [28].

Gibb et al. were the first to investigate the expression level of the lncRNAs in OSCC [29]. Studies have shown HOTAIR was upregulated in OSCC tissues compared to normal adjacent tissues [30, 31]. Additionally, HOTAIR was found to be overexpressed in LSCC tissues [32]. Urothelial cancer-associated 1 (UCA1), which is thought to play a role in bladder cancer progression, was significantly upregulated in TSCC and OSCC [33, 34]. Metastasis-associated lung adenocarcinoma transcript 1 (MALAT1) is another lncRNA upregulated in HNSCC, including LSCC, TSCC, and OSCC [35, 36]. These findings suggest that lncRNAs may be suitable future diagnostic biomarkers in patients with HNSCC.

Therefore, exploring the potential of lncRNAs as diagnostic markers in patients with HNSCC may provide new insights into the disease’s molecular mechanisms and potentially lead to the development of more precise and effective diagnostic approaches. The present systematic review and meta-analysis aimed to investigate the diagnostic accuracy of lncRNAs in individuals with HNSCC.

## Methods

Our systematic review and meta-analysis adhered to the guidelines outlined in the Preferred Reporting Items for Systematic Reviews and Meta-Analyses (PRISMA) Statement [37]. Our systematic review and meta-analysis protocol was registered at PROSPERO with the registration number CRD42023424362.

### Literature search

A comprehensive search was conducted in PubMed, Web of Science (ISI), Scopus, and Embase for English papers until April 1^st^, 2023, with no restrictions on publication year. We utilized a search query for titles, abstracts, and keywords in our chosen databases to perform a systematic search. The search query is available in S1 Table.

### Selection criteria

All peer-reviewed original research that reported sensitivity, specificity, or area under the curve (AUC) values of plasma lncRNAs for diagnosing HNSCC were included.

We included original human studies conducted prospectively or retrospectively on samples obtained from cancer patients with pathological diagnoses and healthy participants. Diagnostic accuracy studies should have compared lncRNAs to an acceptable reference control to determine sensitivity and specificity regardless of test assay time. We considered the studies on tissue bank samples collected from prospectively selected groups to be eligible because they avoided omitting critical data from reporting results. The period between sample collection and laboratory testing may affect test results, which may be affected by sample storage conditions and the stability of each particular biomarker throughout storage and freeze-thawing. Most studies did not have this information readily accessible; thus, we did not examine it in our review. We did not apply eligibility restrictions based on the healthcare settings where the research was conducted and the total number of participants in the included studies.

Non-peer-reviewed and non-English studies, studies using datasets, letters, comments, reviews, case reports, and case series were deemed ineligible and excluded.

After removing duplicates, two authors (SK and MM) screened the titles and abstracts of all identified studies based on the predefined inclusion and exclusion criteria to determine eligibility. After collecting eligible studies, both authors conducted a comprehensive full-text review independently. Any conflicts that arose during the review process were resolved through consensus.

### Data collection

Two reviewers (PF, SK) independently obtained the following data using a standardized extraction: author name, publication year, study design, cancer type, specimen type, sample size, lncRNA name, control population, change in levels of lncRNA in patients compared to the control group, sensitivity, specificity, and area under the curve (AUC) and its 95% confidence interval (CI), and p-value. The third researcher (MM) assessed the probable discrepancies between data extraction files, and any disagreements were resolved by consensus.

### Quality assessment

QUADAS-C was utilized to evaluate the quality of included observational studies [38]. The QUADAS-C tool serves as a method for assessing the potential for bias in studies that compare the accuracy of diagnostic tests. QUADAS-C works by a series of questions to each of the four domains of QUADAS-2: Patient Selection, Index Test, Reference Standard, and Flow and Timing. QUADAS-C provides a set of signaling questions for each question to help guide the answer. The answers can be “yes”, “no”, or “unclear”, depending on the information available in the study report. Based on the answers to the guideline questions, QUADAS-C provides guidance on how to assess the risk of bias for each domain as “low”, “high”, or “unclear”. The overall risk of bias for the comparison can then be assessed by considering the risk of bias for each domain.

### Statistical analysis

We used Reitsma et al.’s (2005) bivariate random effect model to pool studies reporting diagnostic specificity and sensitivity [39]. A bivariate generalized linear mixed model (GLMM) captures the test’s sensitivity, specificity, correlation, and variability across studies. This model uses logit transformation to combine test sensitivity and specificity across multiple studies, taking into account their interdependence. The summary receiver operating characteristic (sROC) curve and AUC, which indicate the test’s precision, were also calculated. The random effects model was used to meta-analyze diagnostic AUC values using the inverse variance method for all studies. This approach was chosen due to the anticipated heterogeneity among the included studies. The approach is capable of computing a summary AUC and its corresponding CI for the aggregated studies.

Diagnostic Odds Ratios reported by some studies were converted to diagnostic AUC using a method introduced by (Salgado, 2018) [40]. The standard error of the AUCs for use in this model was calculated from the 95% CI, if reported, or from the AUC value itself and the sample size. I2 and DerSimonian-Lairdestimator for tau2 statistics were used to assess study heterogeneity. To further explore the heterogeneity, a subgroup analysis was conducted based on the type of specimen obtained. The statistical analyses and visualizations were performed using R version 4.2.2. A statistically significant result was defined as having an I2 value greater than 50% and a p-value less than 0.05.

## Results

### Basic characteristics

**Fig 1:**
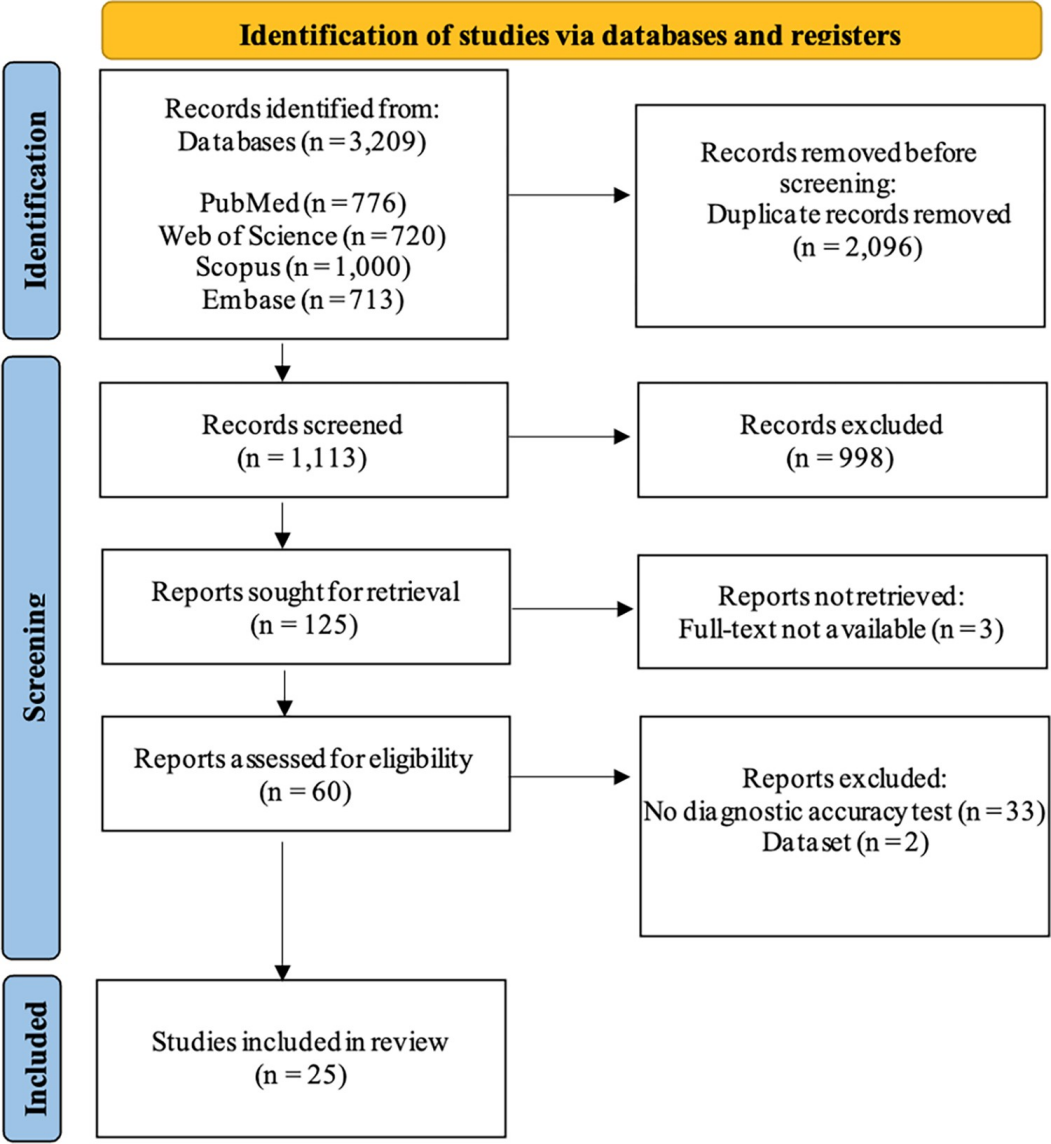
Flow chart of systematic review according to Preferred Reporting Items for Systematic Reviews and Meta-Analysis (PRISMA) guideline.

Upon conducting the primary search of the database, a total of 3209 titles were added. Following the removal of duplicates, the titles and abstracts of 1113 articles were subject to screening for inclusion, with 1043 articles subsequently being excluded. After conducting a thorough review, 63 articles were deemed suitable for full-text analysis. Twenty-five studies fulfilled the criteria for inclusion and 38 studies were excluded (S2 Table). The study selection and exclusion details are delineated by the PRISMA flowchart, as depicted in Fig 1.

Table 1 presents a summary of the basic characteristics of the included studies. The included papers were published between 2014 to 2023. The review included a sample size of 5404 HNSCC cases and 576 healthy controls sourced from various countries, including China, Turkey, Poland, Taiwan, Romania, and Iran. Forty-four diagnostic evaluations were on HNSCC cases of different types, 21 on LSCC, one on hypopharyngeal squamous cell carcinoma (HSCC), 15 on OSCC, and one on TSCC. Three types of specimens were evaluated, including 12 tumor tissue samples, 55 blood samples, and 15 saliva samples. Out of 82 diagnostic evaluations in the included studies, a total of 71 unique lncRNAs were identified. Fifty-seven diagnostic evaluations observed the upregulation of lncRNAs, while 23 observed their downregulation. The diagnostic potential of lncRNA MALAT1 has been validated in five studies. Fifty-two diagnostic evaluations have published sensitivity and specificity metrics lncRNAs to diagnose HNSCC. Each of these 52 evaluations assessed a different type of lncRNA.

**Table 1:** Basic characteristics of the included studies.

ID	Author, year	Country	Study design	Specimen	Sample size	Cancer type	Control type	Case N.	Control N.	lncRNA	Up/down regulation	Sensitivity %	Specificity %	AUC	p value	OR
1	Yao, 2018 [41]	China	case-control	Tumor tissue	40	HNSCC	healthy controls, normal adjacent tissue	20	20	LINC00964	up			0.387 [0.224; 0.55]	0.171	
				Blood	200		serum, healthy controls	100	100	HOXA11-AS	up			0.659 [0.584; 0.735]	0	
					200			100	100	LINC00964	up			0.687 [0.614; 0.759]	0	
					200			100	100	MALAT1	up			0.718 [0.646; 0.79]	0	
					40			20	20	HOXA11-AS	up			0.773 [0.64; 0.905]	0.001	
					40			20	20	MALAT1	up			0.918 [0.838; 0.999]	0	
2	Wu, 2018 [42]	China	case-control	Blood	1098	HNSCC	serum, healthy controls	366	732	rs7958904				0.478 [0.72; 1.04]	0.132	0.87
								366	732	rs874945				0.48 [0.72; 1.09]	0.241	0.88
								366	732	HOTAIR, rs4759314 polymorphism	up			0.53 [1.01; 1.46]	0.043	1.21
3	Aktan, 2022 [43]	Turkey	case-control	Saliva	60	LSCC	saliva (healthy controls)	35	25	LINC02015	down	28.57	92	0.502 [0.353; 0.651]	0.982	
								35	25	LINC02584	down	28.57	92	0.502 [0.353; 0.651]	0.982	
								35	25	TPRG1-AS2	down	88.57	28	0.517 [0.359; 0.674]	0.837	
								35	25	LINC00501	down	45.71	68	0.522 [0.37; 0.674]	0.774	
								35	25	LINC00578	down	37.14	84	0.526 [0.377; 0.675]	0.735	
								35	25	SHANK2-AS3	down	42.86	76	0.535 [0.386; 0.684]	0.647	
								35	25	PEX5L-AS2	down	65.71	65	0.584 [0.432; 0.736]	0.28	
								35	25	SHANK2-AS1	up	91.43	36	0.616 [0.462; 0.77]	0.139	
								35	25	SOX2-OT	down	51.43	76	0.617 [0.47; 0.765]	0.12	
								35	25	ABCC5-AS1	down	48.57	84	0.65 [0.511; 0.79]	0.035	
								35	25	LINC01209	down	77.14	60	0.665 [0.52; 0.81]	0.026	
								35	25	LINC01206	down	51.43	88	0.702 [0.57; 0.833]	0.003	
								35	25	LINC01994	down	82.86	76	0.803 [0.688; 0.919]	<0.001	
4	Lasinska, 2020 [44]	Poland	case-control	Blood	67	HNSCC	serum, healthy controls	53	14	EVF1 & EVF2	up	50	100	0.577	0.024	
								53	14	WT1-AS	up	57.5	71.4	0.593	0.049	
								53	14	mascRNA	up	46.2	100	0.632	0.0045	
								53	14	HOXA6as	up	80.8	71.4	0.637	0.0069	
								53	14	TncRNA	up	34.6	100	0.643	0.01	
								53	14	SNHG4	up	38.5	100	0.659	0.017	
								53	14	Emx2os	up	88.5	57.1	0.703	0.034	
								53	14	Kcnq1ot1	up	69.2	85.7	0.714	0.0062	
								53	14	E2F4 antisense	up	80.8	71.4	0.758	0.0095	
								53	14	RNCR3	up	88.5	57.1	0.758	0.00079	
								53	14	Jpx	up	96.2	57.1	0.769	0.0031	
								53	14	MER11C	up	53.8	100	0.769	0.0007	
								53	14	Dios3os	up	76.9	85.7	0.786	0.008	
								53	14	H19	up	80.8	85.7	0.797	0.0018	
								53	14	YRNA1	up	80.8	71.4	0.797	0.003	
								53	14	SRA	up	88.5	71.4	0.808	0.007	
								53	14	TEA ncRNAs	up	61.5	100	0.808	0.033	
								53	14	SCA8	up	80.8	85.7	0.819	0.0004	
								53	14	ST7OT	up	84.6	71.4	0.841	0.003	
								53	14	21A	up	76.9	85.7	0.846	0.0014	
								53	14	Air	up	61.5	100	0.863	0.000014	
								53	14	BACE1AS	up	73.1	100	0.865	0.001	
								53	14	Alpha 280	up	88.5	85.7	0.874	0.00066	
								53	14	UCA1	up	88.5	85.7	0.879	0.01	
								53	14	Zfas1	up	100	71.4	0.879	0.001	
								53	14	NRON	up	100	71.4	0.885	0.00008	
								53	14	SNHG6	up	73.1	100	0.885	0.002	
								53	14	SNHG3	up	88.5	85.7	0.89	0.00002	
								53	14	ncR-uPAR	up	80.8	85.7	0.896	0.0001	
								53	14	p53 mRNA	up	73.1	100	0.901	0.0068	
								53	14	GAS5	up	76.9	100	0.918	0.00001	
								53	14	SNHG1	up	76.9	100	0.934	0.0017	
								53	14	ANRIL	up	100	100	1	0.0018	
								53	14	lincRNA-RoR	up	100	100	1	0.00025	
5	Shen, 2014 [45]	China	case-control	Tumor tissue	87	LSCC	normal adjacent tissue	87	-	AC026166.2–001	down			0.65	<0.001	
								87	-	RP11-169D4.1–001	down			0.67		
6	Shen, 2019 [46]	China	case-control	Tumor tissue	70	HSCC	normal adjacent tissue	70	-	RP11_169D4.1_001	down	54	70	0.66 [0.568; 0.749]	<0.05	
7	Bozgeyik, 2022 [47]	Turkey	case-control	Tumor tissue	36	OSCC	adjacent normal tissue	36	-	VIM-AS1	up	63	66.67	0.6857 [0.5577; 0.8137]	0.0085	
8	Wang, 2014 [48]	China	case-control	Blood	101	LSCC	vocal cord polyps	52	49	HOTAIR	up	92.3	57.1	0.727 [0.629; 0.811]	<0.000	
9	Shieh, 2021 [49]	Taiwan	cross-sectional	Saliva	102	OSCC	individuals without OSCC	59	43	XIST	down			0.73	<0.001	19.556
					53		females without OSCC	26	27	XIST	down			0.915	<0.001	33.733
10	Aghiorghiesei, 2022 [50]	Romania	cohort	Tumor tissue	33	OSCC	adjacent normal tissue	33	-	H19	down			0.746	<0.001	
								33	-	MALAT1	down			0.7984	<0.01	
11	Zheng, 2020 [51]	China	cohort	Blood	130	OSCC	serum, healthy controls	90	40	SAMMSON	up			0.746	< 0.001	
12	Giragosyan, 2018 [52]	China	case-control	Blood	43	LSCC	serum, healthy controls	22	21	MALAT1	up			0.753 [0.601; 0.905]	0.004	
13	Liu, 2020 [53]	China	Cohort	Tumor tissue	105	LSCC	normal adjacent tissue	105	-	LINC01194	up			0.8388 [0.7839; 0.8937]	< 0.0001	
14	Yuan, 2021 [54]	China	case-control	Tumor tissue	106	OSCC	normal oral mucosal tissue, healthy controls	80	26	LINC01793	up	76.51	83.69	0.84 [0.75; 0.93]	< 0.001	
15	Dong, 2018 [55]	China	case-control	Blood	174	OSCC	serum, healthy controls	122	52	CASC2	down			0.8445 [0.7728; 0.9162]	<0.05	
16	Zhang, 2019 [56]	China	case-control	Blood	120	OSCC	serum, healthy controls	68	52	PAPAS	up			0.85 [0.77; 0.94]		
17	Shaoa, 2018 [57]	China	case-control	Blood	150	OSCC	serum, healthy controls	80	70	AC007271.3	up	77.5	84.5	0.873 [0.815; 0.931]		
18	Cui, 2019 [58]	China	case-control	Blood	110	LSCC	serum, healthy controls	62	48	NEF	down			0.8782 [0.8064; 0.95]	<0.0001	
19	Le, 2020 [59]	China	case-control	Blood	55	OSCC	serum, healthy controls	-	55	NCK1_AS1	up			0.88 [0.83; 0.94]		
					104		serum, OU patients	55	49	NCK1_AS1	up			0.93 [0.88; 0.97]		
20	Zhang, 2019 [60]	China	case-control	Blood	86	OSCC	serum, healthy controls	46	40	CASC15	up			0.88 [0.85; 0.95]		
					86	OSCC	oral ulcer	46	40	CASC15	up			0.91 [0.86; 0.96]		
21	Gao, 2019 [61]	China	case-control	Tumor tissue	94	LSCC	Laryngeal biopsies	66	28	Loc285194	down			0.881 [0.8124; 0.9405]	<0.0001	
22	Sun, 2019 [62]	China	case-control	Blood	180	LSCC	serum, healthy controls	90	90	UCA1	up			0.8905 [0.8408; 0.9402]	<0.0001	
23	Pirhoushiaran, 2021 [63]	Iran	case-control	Tumor tissue	50	HNSCC	normal adjacent tissue	50	-	Fer1L4	down			0.9252	<0.0001	
24	Yuan, 2019 [64]	China	case-control	Blood	97	TSCC	serum, healthy controls	72	25	MALAT1	up			0.9514 [0.9111; 0.9917]	<0.0001	
25	Wang, 2020 [65]	China	case-control	Tumor tissue	228	OSCC	nontumoral tissue	152	76	SNHG1	up			0.974	<0.05	

### Quality assessment

**Fig 2:**
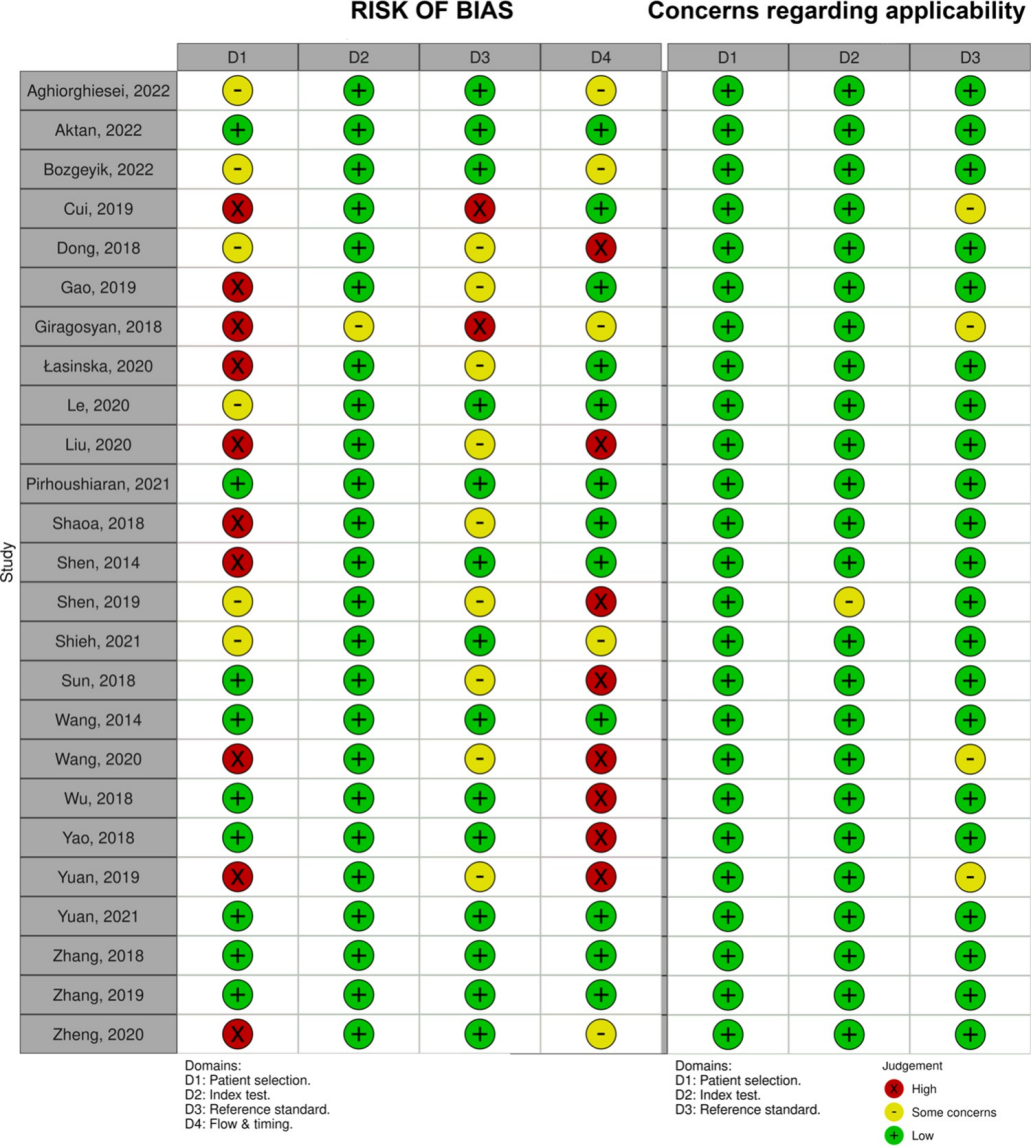
Cochrane’s risk of bias graph for the QUADAS-C tool.

**Fig 3:**
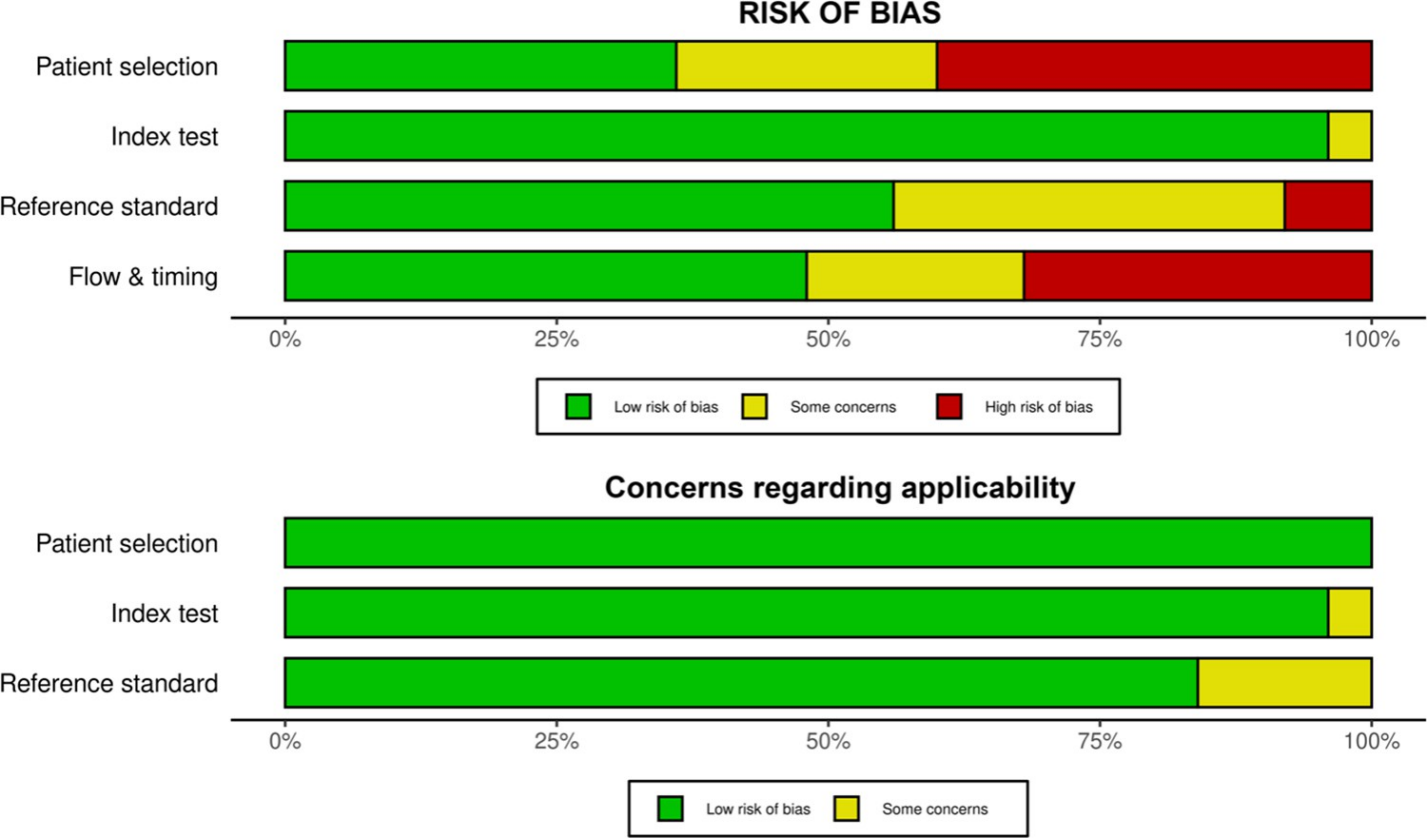
Cochrane’s risk of bias scores for the QUADAS-C tool.

The included studies were evaluated by independent investigators using the QUADAS-C tool for quality assessment. A third assessor was responsible for resolving any discrepancies in quality evaluation. The outcomes of the quality assessment of the incorporated research are illustrated in Figs 2 and 3, which depict Cochrane’s risk of bias graph and the risk of bias scores for each included study, respectively. In 96% of studies, there was a low-risk of bias in the index test domain. In the reference standard and flow & time domains, 56% and 48% of studies, respectively, had a low-risk score, whereas only 36% of studies in the patient selection domain had a low-risk score. Concerns regarding applicability were rated as low risk in all domains across studies.

### Meta-analysis of diagnostic accuracy of lncRNAs in HNSCC

**Fig 4:**
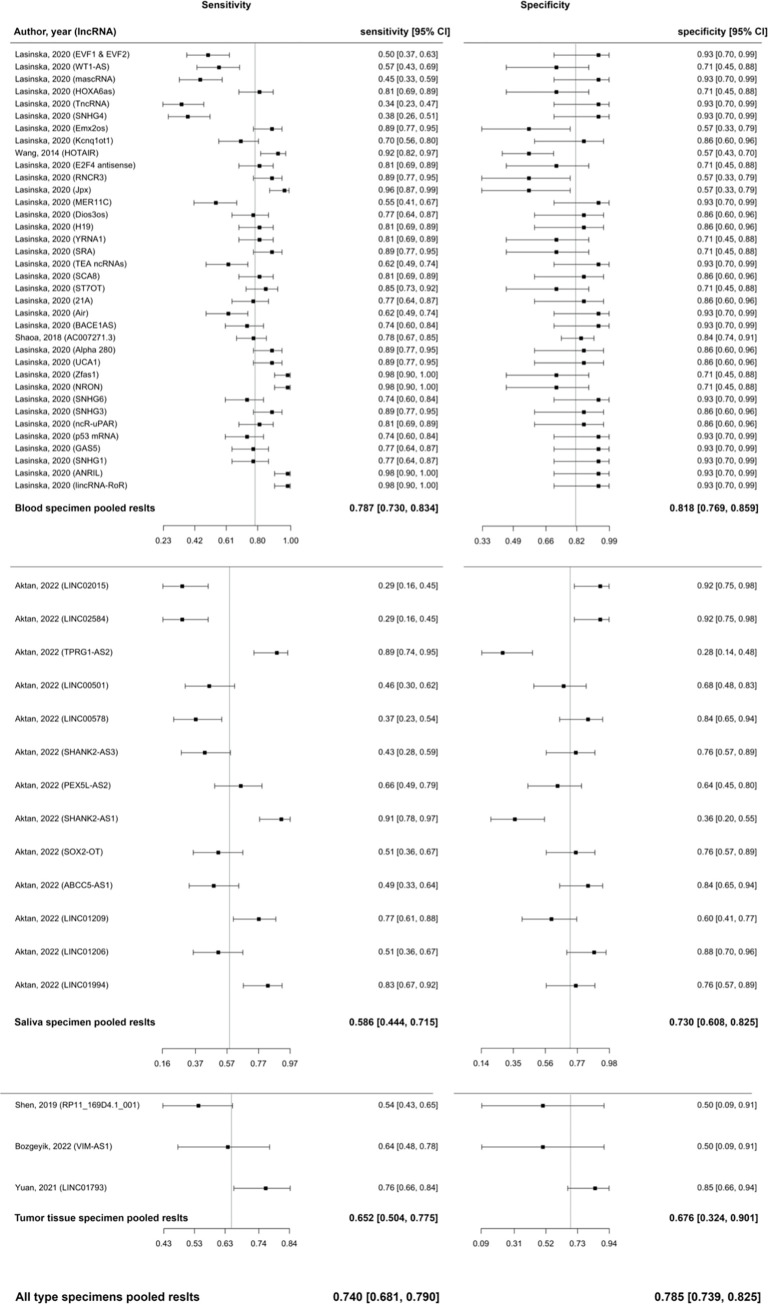
Forest plot for the bivariate model of diagnostic meta-analysis of subgroups.

**Fig 5:**
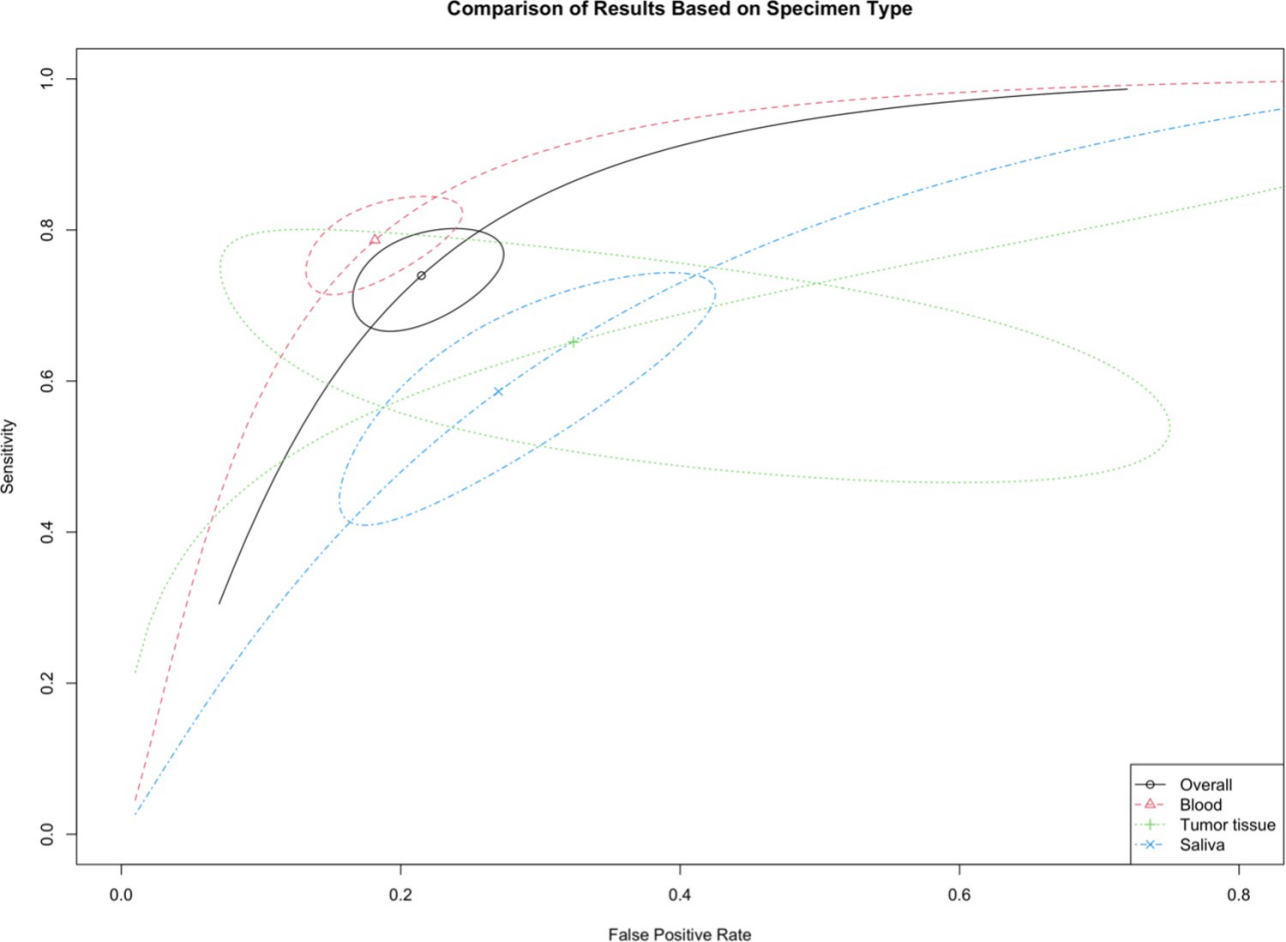
Summary Receiver Operating Characteristic (SROC) curves of subgroups and overall analysis for bivariate model.

The cumulative sensitivity estimated by the Reitsma bivariant model for lncRNAs in the diagnosis of HNSCC was 0.74 (95% CI: 0.68–0.79, p-value = 0.000), and the pooled specificity estimate was 0.79 (95% CI: 0.74–0.83, p-value = 0.000, Fig 4). I2 estimate was 56–66.7%, based on the Holling sample size unadjusted approach. The SROC curve was plotted for each specimen type, and pooled AUC for all types of specimens was found to be 0.83 (Fig 5). As the findings for each subgroup are provided in Table 2, sensitivity, specificity, and AUC for the blood were 0.787, 0.818, and 0.871, respectively. Sensitivity, specificity, and AUC for the salvia, were 0.586, 0.730, and 0.712, respectively; and for tumor tissue were 0.652, 0.676, and 0.698, respectively.

**Table 2:** Bivariate model for diagnostic meta-analysis, estimation method: REML.

Specimen	N	Sensitivity [95% CI]	p-value	Specificity [95% CI]	p-value	AUC	I2 Holling sample size unadjusted approach	X-squared p-value for equality of sensitivities	X-squared p-value for equality of specificities	Correlation of sensitivities and false positive rates
**Blood**	36	0.787 [0.730, 0.834]	0.000***	0.818 [0.769, 0.859]	0.000***	0.871	39.2–48.6%	<2e-16***	0.00193**	0.506
**Saliva**	13	0.586 [0.444, 0.715]	0.234	0.730 [0.608, 0.825]	0.000***	0.712	5–6.1%	1.18e-12***	3.7e-08***	0.837
**Tumor tissue**	3	0.652 [0.504, 0.775]	0.045*	0.676 [0.324, 0.901]	0.327	0.698	47.2–70.9%	0.018*	0.273	NA due to small number of studies

Significancy codes

0 ‘***’

< 0.01 ‘**’

< 0.05 ‘*’

0.1 ‘ ‘; N = number of evaluations, AUC = Area Under the Curves

**Fig 6:**
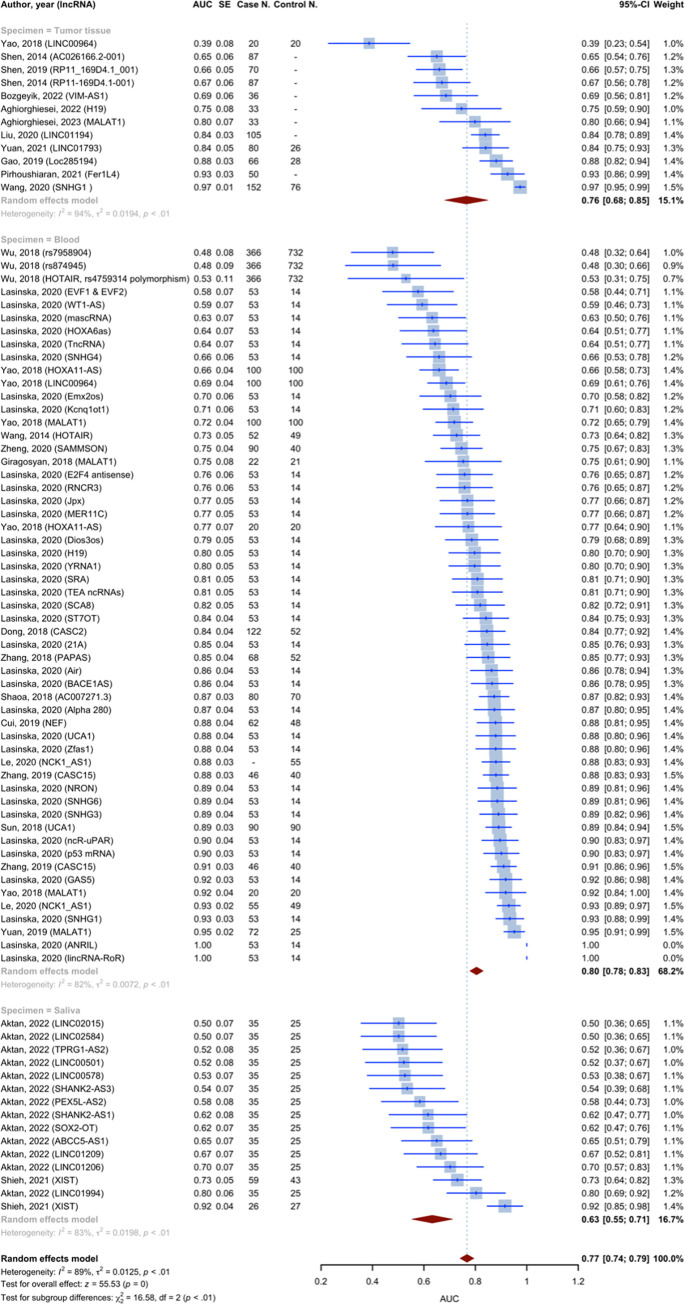
Forest plot for Area Under the Curves (AUCs) of included studies using random effect model with inverse method.

All studies incorporated in the analysis provided AUC values of lncRNAs for detecting HNSCC. The pooled AUC value was 0.77 (95% CI: 0.74–0.79, p-value = 0; I2 = 89.0%, p-value < 0.0001), calculated using the inverse variance method and derived from 82 diagnostic accuracy evaluations and 71 individual lncRNAs (Fig 6). The studies were categorized into different subgroups based on the type of specimen used to measure the expression of lncRNA. The pooled AUC for the blood specimen subgroup, saliva subgroup, and tumor tissue subgroup were 0.8045, 0.6322, and 0.7647, respectively. The findings for each subgroup are provided in Table 3. The test for between subgroup differences was statistically significant (Q = 16.58, p-value = 0.0003).

**Table 3:** Subgroup analysis of the AUS meta-analysis of included studies.

Specimen	N	AUC [95%-CI]	I^2
**Blood**	53	0.8045 [0.7783; 0.8306]	82.0%
**Saliva**	15	0.6322 [0.5529; 0.7116]	82.8%
**Tumor tissue**	12	0.7647 [0.6805; 0.8489]	94.0%

N = number of evaluations, AUC = Area Under the Curves

**Fig 7:**
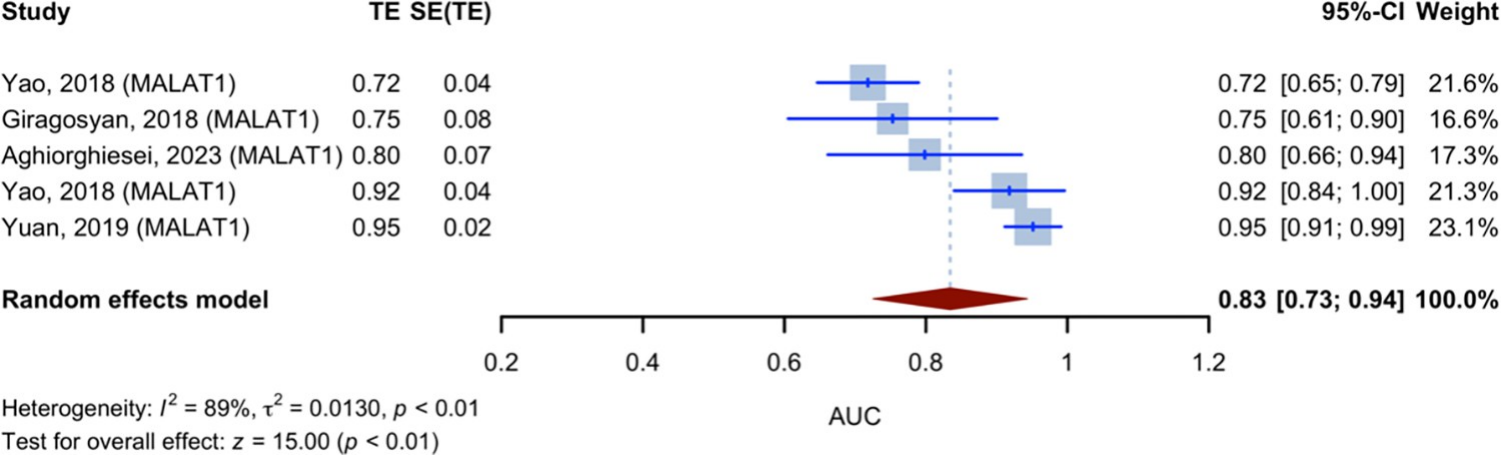
Forest plot for Area Under the Curves (AUCs) of studies evaluated MALAT1 lncRNA using random effect model with inverse method.

Five studies have reported on the diagnostic accuracy of lncRNA MALAT1. Given that the aforementioned studies solely provided AUC values, the inverse variance method was used to calculate the pooled AUC value of these five studies, which was 0.83 (95% CI [0.73; 0.94], p-value 0.0001, I2 = 89.1%, Fig 7).

### Publication bias

**Fig 8:**
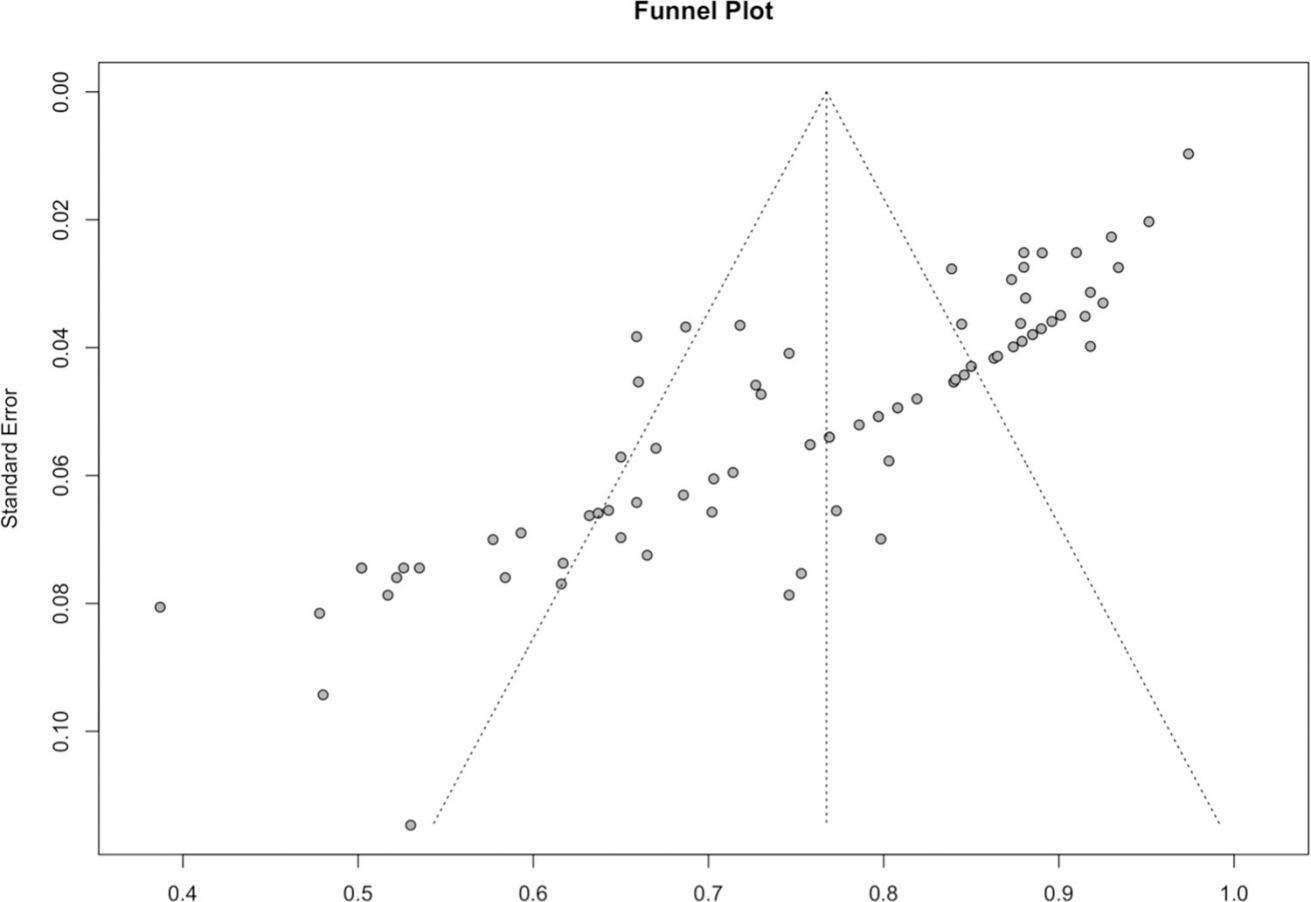
Funnel plot of included studies.

Fig 8 depicts the funnel plot of the included studies in the meta-analysis, which corresponds to the standard error of the AUCs. The asymmetrical funnel plot can indicate the presence of publication bias. Hence, statistical tests, including Begg’s rank correlation test and Egger’s linear regression test, were used to assess potential asymmetry in the funnel plot. According to the findings of the analysis, the studies included showed significant indications of publication bias (Begg’s test p-values < 0.0001 and Eggers’ test p-values < 0.0001).

## Discussion

The present systematic review and meta-analysis evaluated the diagnostic accuracy of lncRNAs in patients with HNSCC. Our study showed that levels of lncRNA in the plasma and tissues of patients with HNSCC could be used as a diagnostic biomarker. We identified 71 unique lncRNAs that were differentially expressed in HNSCC patients compared to healthy controls. The most frequently validated lncRNA was MALAT1, upregulated in five studies with a pooled AUC of 0.83. In 52 diagnostic evaluations, the pooled sensitivity and specificity of lncRNAs for diagnosing HNSCC were 0.74 and 0.79, respectively, with a pooled AUC of 0.83 across all specimen types. The specimen type significantly impacted the diagnostic accuracy of lncRNAs, with blood having the highest AUC of 0.871 and the tumor tissue having the lowest AUC of 0.698. Eighty-two diagnostic accuracy evaluations and 71 lncRNAs yielded a pooled AUC of 0.77. The AUCs for blood and tumor tissue subgroups were 0.8045 and 0.7647, respectively.

LncRNAs are RNA transcripts that exceed 200 nucleotides and do not encode proteins or peptides. They regulate gene expression and function at the transcriptional, translational, and post-translational levels, thereby exerting diverse biological functions. In the past decade, it has become evident that dysregulated lncRNA profiles are involved in the pathogenesis of numerous diseases, including cancer. Specifically, lncRNAs play a critical role in tumor growth and metastasis and have been identified as potential biomarkers and targets for cancer diagnosis and treatment [66].

Despite continuous research to develop novel treatments and improve knowledge of the mechanisms driving tumor formation, treating cancers, especially those diagnosed in later stages with a dismal prognosis, remains challenging. The substantial fatality rate linked to cancer is partly attributed to inadequate early detection techniques and/or imprecise diagnostic tools, including certain protein biomarkers. As lncRNAs have high stability in the bloodstream and are resistant to nuclease-mediated degradation, they are considered more reliable than other circulating nucleic acids. Their abundance and stability in circulation make them promising cancer biomarkers compared to other analytes, such as circulating tumor cells, cell-free nucleic acids (including ctDNA), and exosomes. Although the diagnostic performance of individual circulating lncRNAs is relatively poor, to improve their specificity and sensitivity, several studies have combined the diagnostic values of multiple circulating lncRNAs [67].

With a growing interest in exploring the function of lncRNAs in different types of cancers, researchers have discovered various lncRNAs that are differentially expressed in HNSCC cell lines. LncRNAs, such as CASC9 [68], FTH1P3 [69], ZFAS1 [70], LINC01929 [71], MEG3 [72], HCG18 [73], etc., were found to be dysregulated in HNSCC cell lines. Moreover, bioinformatics analysis showed that lncRNAs are differentially expressed in patients with HNSCC, and their expression levels could be associated with disease stage, survival, and drug resistance [74].

LncRNAs could also be used as prognostic and response to therapy biomarkers in patients with HNSCC. Mutations or dysregulated expression of lncRNAs like POP1-1 [75], EGFR-AS1 [76], GAS5 [77], BARX1-DT, KLHL7-DT, and LINC02154 [78], seem to be associated with response to different types of therapies, such as chemotherapy, chemoradiotherapy, and immunotherapy in patients with HNSCC. Ferroptosis-associated lncRNAs [79], MALAT1, HOTAIR, HOTTIP, LINC02487, LINC00511 [80], PHACTR2-AS1 [81], and CEBPA-AS1 [82] are examples of lncRNAs associated with poor prognosis in patients with HNSCC. Additionally, using more than one type of lncRNAs as a signature for the prognosis prediction could increase their accuracy as a biomarker [83].

LncRNAs have been discovered to have an important role in controlling several aspects of HNSCC pathogenesis, including but not limited to proliferation, survival, metastasis, treatment resistance, immunological response, and angiogenesis [8, 84]. Modulation of many signaling pathways in HNSCC has been found to be related to lncRNAs, including but not limited to Wnt/β-catenin, PI3K/AKT/mTOR, JAK/STST3, p53, NF-B, TGF-β/Smad, and Notch [85–88]. Moreover, there is evidence suggesting that the pairing of lncRNAs with messenger RNAs (mRNAs) is associated with various biological processes, including regulation of transcription, macromolecule synthesis, immunological cells synapse development, and immunological signaling pathways such as B and T cell signaling and the signaling involving TGF-β receptor [8]. According to one study, increased levels of LINC00460 were connected with cancer-related molecular pathways such as EMT and other inflammatory response pathways [89]. LncRNAs can interact with DNA, RNA, or proteins to influence gene expression at either the transcriptional or post-transcriptional levels. LncRNAs have been shown to act as decoys, scaffolding, guides, or sponges for their targets [8, 84]. LncRNAs can form complex networks with other lncRNAs or microRNAs, influencing the course of HNSCC. LncRNAs are subject to a variety of influences, including but not limited to hypoxia, oxidative stress, copper metabolism, and HPV infection, all of which have the potential to modulate their expression and functional roles in HNSCC [84].

MALAT1 is a lncRNA involved in various biological processes and can influence the onset and progression of several malignant cancers, including HNSCC. MALAT1 has the potential to influence HNSCC cell proliferation, migration, invasion, apoptosis, angiogenesis, drug resistance, and immunological response by mechanisms including transcriptional control, epigenetic alteration, microRNA sponge, RNA binding protein interaction, and chromatin remodeling [35, 90, 91]. MALAT1 expression in HNSCC tissues and cells is frequently dysregulated, and its level can be affected by variables such as HPV infection, smoking, alcohol intake, and hypoxia. MALAT1 has the potential to be a therapeutic target for HNSCC therapy. Several studies have found that suppressing or silencing MALAT1 can limit HNSCC growth and metastasis while also increasing HNSCC cell susceptibility to radiation and chemotherapy. However, the precise role and mechanism of MALAT1 in HNSCC are still unknown, and additional study is required to understand its function and clinical importance in HNSCC [90, 92].

LncRNAs have different expression patterns that are particular to the individual’s tissue and condition, making them attractive candidates for use as biomarkers and therapeutic targets in the setting of HNSCC. LncRNAs can also be found in a range of bodily fluids, supporting their potential utility in this area [8, 84–86].

Our study’s use of meta-analysis is a valuable approach for evaluating the diagnostic precision of lncRNA in the context of HNSCC. The ability to combine data from multiple studies, increasing sample size, and statistical power to detect minor or moderate effects of lncRNA as diagnostic biomarkers are all advantages of such large-scale reviews of previous studies. It has the potential to reduce the impact of stochastic errors and partialities in single studies, resulting in more precise and reliable diagnostic precision assessments. Statistical techniques were used in this study to investigate the underlying causes of study heterogeneity and inconsistency. Furthermore, we attempted to identify potential moderators or confounders that could affect the diagnostic performance of lncRNA. Despite its benefits, meta-analysis is not without limitations. If the studies used are not sufficiently comprehensive, representative, or rigorous, it may be subject to publication bias, selection bias, or quality bias. When attempting to combine data from various study designs, methodologies, populations, or outcomes, the meta-analysis process may encounter difficulties. This may introduce variability and uncertainty into the final results. It is possible that the meta-analysis did not take into account all of the variables that influence the diagnostic precision of lncRNA, including but not limited to lncRNA characteristics such as type, expression, function, and regulation, detection techniques and platforms, criteria and cut-off values used, and the clinical context and scenarios in which the analysis is performed.

Several variables continue to restrict the quality of data on lncRNAs as diagnostic biomarkers for HNSCC. To begin, lncRNA expression levels might differ based on tissue or cell type, sample collection method, storage conditions, and detection technology. To guarantee the reliability and repeatability of lncRNA measurements, defined techniques and quality control measures are required. Second, little is known about the biological activities and processes of lncRNAs in HNSCC. More research is needed to determine how lncRNAs contribute to the beginning and development of HNSCC, as well as how they interact with other molecules or pathways. Third, the clinical value and validity of lncRNAs as diagnostic biomarkers for HNSCC must be investigated further in large-scale prospective trials with well-defined populations and objectives. lncRNAs’ sensitivity, specificity, accuracy, and predictive value should be compared to existing biomarkers or clinical indicators.

Several variables influence the application of data on lncRNAs as diagnostic biomarkers for HNSCC. First, the specificity and sensitivity of lncRNAs for HNSCC should be high enough to differentiate it from other forms of cancer or benign disorders affecting the head and neck area. Second, lncRNA stability and availability in biological samples should be adequate to enable simple and accurate identification and quantification. Third, the cost-effectiveness and practicality of lncRNA-based testing should be equivalent to or better than current techniques or standards. Fourth, large-scale prospective studies with well-defined cohorts and objectives should be conducted to evaluate the clinical relevance and efficacy of lncRNAs for HNSCC.

## Conclusion

The findings of our study suggest that lncRNAs could potentially function as effective diagnostic biomarkers for cases of HNSCC. This could enhance the current diagnostic methods and provide further understanding of the personalized treatment and management of HNSCC patients. Additional investigation is necessary to validate the clinical efficacy of lncRNAs in a broader range of patients with HNSCC, as well as to elucidate the underlying mechanisms through which lncRNAs participate in the pathogenesis of HNSCC. Furthermore, further research is required to discover novel lncRNAs associated with the growth and advancement of HNSCC. Subsequent investigations ought to utilize high-throughput sequencing methodologies and bioinformatics software to ascertain the expression profiles and signatures of lncRNAs in both lymph node tissues and circulating fluids.

## Supporting information

S1 Table Search strategy for each database. (DOCX)

S2 Table Excluded studies. (DOCX)

S3 Table PRISMA 2020 checklist. (DOCX)
