# Supplementary material for: Long non-coding RNA as a potential diagnostic biomarker in head and neck squamous cell carcinoma: A systematic review and meta-analysis
Source: PLoS One. 2023 Sep 21;18(9):e0291921. doi: 10.1371/journal.pone.0291921 (PMC10513217; doi:10.1371/journal.pone.0291921)
Supplement: S1 Table — (DOCX) [file pone.0291921.s001.docx]

**Supplementary Table 1.** Search strategy for each database

| **Query** |
| --- |
| **PubMed** |
| (Squamous Cell Carcinoma of Head and Neck[MeSH] OR “Squamous Cell Carcinoma of Head and Neck”[tiab] OR “Head And Neck Squamous Cell Carcinomas”[tiab] OR “Squamous Cell Carcinoma, Head And Neck”[tiab] OR “Squamous Cell Carcinoma of the Head and Neck”[tiab] OR “Head and Neck Squamous Cell Carcinoma”[tiab] OR “HNSCC”[tiab] OR “Carcinoma, Squamous Cell of Head and Neck”[tiab] OR “Squamous Cell Carcinoma of the Larynx”[tiab] OR “Laryngeal Squamous Cell Carcinoma”[tiab] OR “Squamous Cell Carcinoma of Larynx”[tiab] OR “Squamous Cell Carcinoma of the Nasal Cavity”[tiab] OR “Oral Tongue Squamous Cell Carcinoma”[tiab] OR “Hypopharyngeal Squamous Cell Carcinoma”[tiab] OR “Oral Squamous Cell Carcinoma”[tiab] OR “Oral Cavity Squamous Cell Carcinoma”[tiab] OR “Oral Squamous Cell Carcinomas”[tiab] OR “Squamous Cell Carcinoma of the Mouth”[tiab] OR “Oropharyngeal Squamous Cell Carcinoma”[tiab]) AND (“Noncoding RNA, Long”[tiab] OR “lncRNA”[tiab] OR “Long ncRNA”[tiab] OR “ncRNA, Long”[tiab] OR “RNA, Long Non-Translated”[tiab] OR “Long Non-Translated RNA”[tiab] OR “Non-Translated RNA, Long”[tiab] OR “RNA, Long Non Translated”[tiab] OR “Long Non-Coding RNA”[tiab] OR “Long Non Coding RNA”[tiab] OR “Non-Coding RNA, Long”[tiab] OR “RNA, Long Non-Coding”[tiab] OR “Long Non-Protein-Coding RNA”[tiab] OR “Long Non Protein Coding RNA”[tiab] OR “Non-Protein-Coding RNA, Long”[tiab] OR “RNA, Long Non-Protein-Coding”[tiab] OR “Long Noncoding RNA”[tiab] OR “RNA, Long Untranslated”[tiab] OR “Long Untranslated RNA”[tiab] OR “Untranslated RNA, Long”[tiab] OR “Long ncRNAs”[tiab] OR “ncRNAs, Long”[tiab] OR “Long Intergenic Non-Protein Coding RNA”[tiab] OR “Long Intergenic Non Protein Coding RNA”[tiab] OR “LincRNAs”[tiab] OR “LINC RNA”[tiab] OR “LincRNA”[tiab] OR RNA, Long Noncoding[MeSH]) |
| **ISI** |
| TS=(“Squamous Cell Carcinoma of Head and Neck” OR “Head And Neck Squamous Cell Carcinomas” OR “Squamous Cell Carcinoma, Head And Neck” OR “Squamous Cell Carcinoma of the Head and Neck” OR “Head and Neck Squamous Cell Carcinoma” OR “HNSCC” OR “Carcinoma, Squamous Cell of Head and Neck” OR “Squamous Cell Carcinoma of the Larynx” OR “Laryngeal Squamous Cell Carcinoma” OR “Squamous Cell Carcinoma of Larynx” OR “Squamous Cell Carcinoma of the Nasal Cavity” OR “Oral Tongue Squamous Cell Carcinoma” OR “Hypopharyngeal Squamous Cell Carcinoma” OR “Oral Squamous Cell Carcinoma” OR “Oral Cavity Squamous Cell Carcinoma” OR “Oral Squamous Cell Carcinomas” OR “Squamous Cell Carcinoma of the Mouth” OR “Oropharyngeal Squamous Cell Carcinoma”)  AND  TS=(“Noncoding RNA, Long” OR “lncRNA” OR “Long ncRNA” OR “ncRNA, Long” OR “RNA, Long Non-Translated” OR “Long Non-Translated RNA” OR “Non-Translated RNA, Long” OR “RNA, Long Non Translated” OR “Long Non-Coding RNA” OR “Long Non Coding RNA” OR “Non-Coding RNA, Long” OR “RNA, Long Non-Coding” OR “Long Non-Protein-Coding RNA” OR “Long Non Protein Coding RNA” OR “Non-Protein-Coding RNA, Long” OR “RNA, Long Non-Protein-Coding” OR “Long Noncoding RNA” OR “RNA, Long Untranslated” OR “Long Untranslated RNA” OR “Untranslated RNA, Long” OR “Long ncRNAs” OR “ncRNAs, Long” OR “Long Intergenic Non-Protein Coding RNA” OR “Long Intergenic Non Protein Coding RNA” OR “LincRNAs” OR “LINC RNA” OR “LincRNA”) |
| **Scopus** |
| TITLE-ABS-KEY (“Squamous Cell Carcinoma of Head and Neck” OR “Head And Neck Squamous Cell Carcinomas” OR “Squamous Cell Carcinoma, Head And Neck” OR “Squamous Cell Carcinoma of the Head and Neck” OR “Head and Neck Squamous Cell Carcinoma” OR “HNSCC” OR “Carcinoma, Squamous Cell of Head and Neck” OR “Squamous Cell Carcinoma of the Larynx” OR “Laryngeal Squamous Cell Carcinoma” OR “Squamous Cell Carcinoma of Larynx” OR “Squamous Cell Carcinoma of the Nasal Cavity” OR “Oral Tongue Squamous Cell Carcinoma” OR “Hypopharyngeal Squamous Cell Carcinoma” OR “Oral Squamous Cell Carcinoma” OR “Oral Cavity Squamous Cell Carcinoma” OR “Oral Squamous Cell Carcinomas” OR “Squamous Cell Carcinoma of the Mouth” OR “Oropharyngeal Squamous Cell Carcinoma”) AND TITLE-ABS-KEY(“Noncoding RNA, Long” OR “lncRNA” OR “Long ncRNA” OR “ncRNA, Long” OR “RNA, Long Non-Translated” OR “Long Non-Translated RNA” OR “Non-Translated RNA, Long” OR “RNA, Long Non Translated” OR “Long Non-Coding RNA” OR “Long Non Coding RNA” OR “Non-Coding RNA, Long” OR “RNA, Long Non-Coding” OR “Long Non-Protein-Coding RNA” OR “Long Non Protein Coding RNA” OR “Non-Protein-Coding RNA, Long” OR “RNA, Long Non-Protein-Coding” OR “Long Noncoding RNA” OR “RNA, Long Untranslated” OR “Long Untranslated RNA” OR “Untranslated RNA, Long” OR “Long ncRNAs” OR “ncRNAs, Long” OR “Long Intergenic Non-Protein Coding RNA” OR “Long Intergenic Non Protein Coding RNA” OR “LincRNAs” OR “LINC RNA” OR “LincRNA”) |
| **EMBASE** |
| (“Squamous Cell Carcinoma of Head and Neck”:ti,ab,kw OR “Head And Neck Squamous Cell Carcinomas”:ti,ab,kw OR “Squamous Cell Carcinoma, Head And Neck”:ti,ab,kw OR “Squamous Cell Carcinoma of the Head and Neck”:ti,ab,kw OR “Head and Neck Squamous Cell Carcinoma”:ti,ab,kw OR “HNSCC”:ti,ab,kw OR “Carcinoma, Squamous Cell of Head and Neck”:ti,ab,kw OR “Squamous Cell Carcinoma of the Larynx”:ti,ab,kw OR “Laryngeal Squamous Cell Carcinoma”:ti,ab,kw OR “Squamous Cell Carcinoma of Larynx”:ti,ab,kw OR “Squamous Cell Carcinoma of the Nasal Cavity”:ti,ab,kw OR “Oral Tongue Squamous Cell Carcinoma”:ti,ab,kw OR “Hypopharyngeal Squamous Cell Carcinoma”:ti,ab,kw OR “Oral Squamous Cell Carcinoma”:ti,ab,kw OR “Oral Cavity Squamous Cell Carcinoma”:ti,ab,kw OR “Oral Squamous Cell Carcinomas”:ti,ab,kw OR “Squamous Cell Carcinoma of the Mouth”:ti,ab,kw OR “Oropharyngeal Squamous Cell Carcinoma”:ti,ab,kw) AND (“Noncoding RNA, Long”:ti,ab,kw OR “lncRNA”:ti,ab,kw OR “Long ncRNA”:ti,ab,kw OR “ncRNA, Long”:ti,ab,kw OR “RNA, Long Non-Translated”:ti,ab,kw OR “Long Non-Translated RNA”:ti,ab,kw OR “Non-Translated RNA, Long”:ti,ab,kw OR “RNA, Long Non Translated”:ti,ab,kw OR “Long Non-Coding RNA”:ti,ab,kw OR “Long Non Coding RNA”:ti,ab,kw OR “Non-Coding RNA, Long”:ti,ab,kw OR “RNA, Long Non-Coding”:ti,ab,kw OR “Long Non-Protein-Coding RNA”:ti,ab,kw OR “Long Non Protein Coding RNA”:ti,ab,kw OR “Non-Protein-Coding RNA, Long”:ti,ab,kw OR “RNA, Long Non-Protein-Coding”:ti,ab,kw OR “Long Noncoding RNA”:ti,ab,kw OR “RNA, Long Untranslated”:ti,ab,kw OR “Long Untranslated RNA”:ti,ab,kw OR “Untranslated RNA, Long”:ti,ab,kw OR “Long ncRNAs”:ti,ab,kw OR “ncRNAs, Long”:ti,ab,kw OR “Long Intergenic Non-Protein Coding RNA”:ti,ab,kw OR “Long Intergenic Non Protein Coding RNA”:ti,ab,kw OR “LincRNAs”:ti,ab,kw OR “LINC RNA”:ti,ab,kw OR “LincRNA”:ti,ab,kw) |
