# Supplementary material for: Long non-coding RNA as a potential diagnostic biomarker in head and neck squamous cell carcinoma: A systematic review and meta-analysis
Source: PLoS One. 2023 Sep 21;18(9):e0291921. doi: 10.1371/journal.pone.0291921 (PMC10513217; doi:10.1371/journal.pone.0291921)
Supplement: S2 Table — (DOCX) [file pone.0291921.s002.docx]

**Supplementary Table 2.** Excluded Studies

| **No.** | **Author, year** | **Title** | **Reason of exclusion** |
| --- | --- | --- | --- |
|  | A. Herreros-Pomares [1], 2021 | Differentially methylated genes in proliferative verrucous leukoplakia reveal potential malignant biomarkers for oral squamous cell carcinoma | No test for diagnostic value |
|  | C. Z. Zhang [2], 2017 | Long intergenic non-coding RNA 668 regulates VEGFA signaling through inhibition of miR-297 in oral squamous cell carcinoma | No test for diagnostic value |
|  | Colombo [3], 2009 | Gene expression profiling reveals molecular marker candidates of laryngeal squamous cell carcinoma | No test for diagnostic value |
|  | D. Xu [4], 2019 | Long non-coding RNA LINC00662 promotes proliferation and migration in oral squamous cell carcinoma | No test for diagnostic value |
|  | F. Song [5], 2021 | Long non‑coding RNA MIAT promotes the proliferation and invasion of laryngeal squamous cell carcinoma cells by sponging microRNA‑613 | No test for diagnostic value |
|  | F. Zhao [6], 2022 | MIR4435-2HG: A Tumor-associated Long Non-coding RNA | Full-text not available |
|  | G. Grubelnik [7], 2021 | MicroRNAs and Long Non-Coding RNAs as Regulators of NANOG Expression in the Development of Oral Squamous Cell Carcinoma | No test for diagnostic value |
|  | H. Feng [8], 2020 | Long non-coding RNA SLC16A1-AS1: its multiple tumorigenesis features and regulatory role in cell cycle in oral squamous cell carcinoma | No test for diagnostic value |
|  | H. Tang [9], 2013 | Salivary lncRNA as a potential marker for Oral squamous cell carcinoma diagnosis | No test for diagnostic value |
|  | J. C. Zhou [10], 2018 | Anti-tumor effect of HOTAIR-miR-613-SNAI2 axis through suppressing EMT and drug resistance in laryngeal squamous cell carcinoma | No test for diagnostic value |
|  | J. Fan [11], 2022 | LncRNA LEF1-AS1 Acts as a Novel Biomarker and Promotes Hypopharyngeal Squamous Cell Carcinoma Progression and Metastasis by Targeting the miR-221-5p/GJA1 Axis | Dataset |
|  | J. Jin [12], 2020 | Bioinformatics analysis of aberrantly expressed exosomal lncRNAs in oral squamous cell carcinoma (CAL.27 vs. oral epithelial) cells | No test for diagnostic value |
|  | J. Liu [13], 2022 | Long Noncoding RNA LINC00941 Promotes Cell Proliferation and Invasion by Interacting with hnRNPK in Oral Squamous Cell Carcinoma | No test for diagnostic value |
|  | J. Wang [14], 2022 | Aberrant methylation-mediated downregulation of the LINC01554 gene accelerates the malignant progression and regulates the chemosensitivity of laryngeal squamous cell carcinoma | No test for diagnostic value |
|  | J. Wu [15], 2015 | Expression of long noncoding RNA-HOX transcript antisense intergenic RNA in oral squamous cell carcinoma and effect on cell growth | No test for diagnostic value |
|  | J. Xu [16], 2020 | lncRNA HOXA11-AS promotes proliferation and migration via sponging miR-155 in hypopharyngeal squamous cell carcinoma | No test for diagnostic value |
|  | J. Yang [17], 2021 | Glycolysis reprogramming in cancer-associated fibroblasts promotes the growth of oral cancer through the lncRNA H19/miR-675-5p/PFKFB3 signaling pathway | No test for diagnostic value |
|  | J. Zhou [18], 2015 | Gene microarray analysis of lncRNA and mRNA expression profiles in patients with hypopharyngeal squamous cell carcinoma | No test for diagnostic value |
|  | Jia [19], 2020 | Screening and validation of plasma long non-coding RNAs as biomarkers for the early diagnosis and staging of oral squamous cell carcinoma | No test for diagnostic value |
|  | K. Lyu [20], 2020 | Using RNA sequencing to identify a putative lncRNA-associated ceRNA network in laryngeal squamous cell carcinoma | No test for diagnostic value |
|  | L. Liu [21], 2020 | LncRNA FGD5-AS1 can be predicted as therapeutic target in oral cancer | No test for diagnostic value |
|  | L. M. Zhang [22], 2018 | Long non-coding RNA ANRIL promotes tumorgenesis through regulation of FGFR1 expression by sponging miR-125a-3p in head and neck squamous cell carcinoma | No test for diagnostic value |
|  | L. Pan [23], 2019 | Long non-coding RNA CASC2 serves as a ceRNA of microRNA-21 to promote PDCD4 expression in oral squamous cell carcinoma | No test for diagnostic value |
|  | M. Sassenberg [24], 2019 | Upregulation of the long non-coding RNA CASC9 as a biomarker for squamous cell carcinoma | Dataset |
|  | M. Sun [25], 2020 | Knockdown of long non-coding RNA (lncRNA) colon cancer-associated transcript-1 (CCAT1) suppresses oral squamous cell carcinoma proliferation, invasion, and migration by inhibiting the discoidin domain receptor 2 (DDR2)/ERK/AKT axis | No test for diagnostic value |
|  | Q. Wang [26], 2021 | Silencing of lncrna snhg16 downregulates cyclin d1 (Ccnd1) to abrogate malignant phenotypes in oral squamous cell carcinoma (oscc) through upregulating mir-17-5p | No test for diagnostic value |
|  | Q. Wang [27], 2021 | LncRNA PVT1 participates in the development of oral squamous cell carcinomas through accelerating EMT and serves as a diagnostic biomarker | Full-text not available |
|  | S. Garo Kyurkchiyan [28], 2020 | Novel insights into laryngeal squamous cell carcinoma from association study of aberrantly expressed miRNAs, lncRNAs and clinical features in Bulgarian patients | No test for diagnostic value |
|  | S. Saproo [29], 2023 | Salivary protein kinase C alpha and novel microRNAs as diagnostic and therapeutic resistance markers for oral squamous cell carcinoma in Indian cohorts | No test for diagnostic value |
|  | S. Zhang [30], 2022 | Long non-coding RNA LINC01296 promotes progression of oral squamous cell carcinoma through activating the MAPK/ERK signaling pathway via the miR-485-5p/PAK4 axis | No test for diagnostic value |
|  | S. Zhou [31], 2021 | Exosome-derived long non-coding RNA ADAMTS9-AS2 suppresses progression of oral submucous fibrosis via AKT signalling pathway | No test for diagnostic value |
|  | T. Kolenda [32], 2016 | LncRNAs as new biomarkers in head and neck cancers | Full-text not available |
|  | Wei Gao [33], 2016 | Potential biomarkers and their regulatory relationships in laryngeal squamous cell carcinoma with lymph node metastasis revealed by integrating mRNA, microRNA and long non-coding RNA profiles | No test for diagnostic value |
|  | X. Qiu [34], 2019 | Knockdown of lncRNA RHPN1-AS1 inhibits cell migration, invasion and proliferation in head and neck squamous cell carcinoma | No test for diagnostic value |
|  | Y. F. Yang [35], 2020 | Silencing novel long non-coding RNA FKBP9P1 represses malignant progression and inhibits PI3K/AKT signaling of head and neck squamous cell carcinoma in vitro | No test for diagnostic value |
|  | Y. T. Yang [36], 2016 | Long non-coding RNA UCA1 contributes to the progression of oral squamous cell carcinoma by regulating the WNT/β-catenin signaling pathway | No test for diagnostic value |
|  | Y. Yang [37], 2019 | Increased expression of lncRNA CASC9 promotes tumor progression by suppressing autophagy-mediated cell apoptosis via the AKT/mTOR pathway in oral squamous cell carcinoma | No test for diagnostic value |
|  | Y. Zhao [38], 2021 | Long non-coding RNA HOXA-AS3 promotes cell proliferation of oral squamous cell carcinoma through sponging microRNA miR-218-5p | No test for diagnostic value |

1. Herreros-Pomares A, Llorens C, Soriano B, Bagan L, Moreno A, Calabuig-Fariñas S, et al. Differentially methylated genes in proliferative verrucous leukoplakia reveal potential malignant biomarkers for oral squamous cell carcinoma. Oral Oncology. 2021;116.

2. Zhang CZ. Long intergenic non-coding RNA 668 regulates VEGFA signaling through inhibition of miR-297 in oral squamous cell carcinoma. Biochem Biophys Res Commun. 2017;489(4):404-12.

3. Colombo J, Fachel AA, Calmon MD, Cury PM, Fukuyama EE, Tajara EH, et al. Gene expression profiling reveals molecular marker candidates of laryngeal squamous cell carcinoma. ONCOLOGY REPORTS. 2009;21(3):649-63.

4. Xu D, Chen Y, Yuan C, Zhang S, Peng W. Long non-coding RNA LINC00662 promotes proliferation and migration in oral squamous cell carcinoma. OncoTargets and Therapy. 2019;12:647-56.

5. Song F, Yang Y, Liu J. Long non‑coding RNA MIAT promotes the proliferation and invasion of laryngeal squamous cell carcinoma cells by sponging microRNA‑613. Experimental and Therapeutic Medicine. 2021;21(3).

6. Zhao F, Liu Y, Tan F, Tang L, Du Z, Mou J, et al. MIR4435-2HG: A Tumor-associated Long Non-coding RNA. Current Pharmaceutical Design. 2022;28(25):2043-51.

7. Grubelnik G, Boštjančič E, Aničin A, Dovšak T, Zidar N. MicroRNAs and Long Non-Coding RNAs as Regulators of NANOG Expression in the Development of Oral Squamous Cell Carcinoma. Frontiers in Oncology. 2021;10.

8. Feng H, Zhang X, Lai W, Wang J. Long non-coding RNA SLC16A1-AS1: its multiple tumorigenesis features and regulatory role in cell cycle in oral squamous cell carcinoma. Cell Cycle. 2020;19(13):1641-53.

9. Tang H, Wu Z, Zhang J, Su B. Salivary lncRNA as a potential marker for Oral squamous cell carcinoma diagnosis. Molecular Medicine Reports. 2013;7(3):761-6.

10. Zhou JC, Zhang JJ, Ma W, Zhang W, Ke ZY, Ma LG. Anti-tumor effect of HOTAIR-miR-613-SNAI2 axis through suppressing EMT and drug resistance in laryngeal squamous cell carcinoma. RSC Adv. 2018;8(52):29879-89.

11. Fan J, Wang C, Zhai X, Li J, Ju J, Zhu Y, et al. LncRNA LEF1-AS1 Acts as a Novel Biomarker and Promotes Hypopharyngeal Squamous Cell Carcinoma Progression and Metastasis by Targeting the miR-221-5p/GJA1 Axis. Disease Markers. 2022;2022.

12. Jin J, Huang Z, Lu X, Wu S, Jia M, Li X, et al. Bioinformatics analysis of aberrantly expressed exosomal lncRNAs in oral squamous cell carcinoma (CAL.27 vs. oral epithelial) cells. Oncology Letters. 2020;20(3):2378-86.

13. Liu J, Li Z, Zhang T, Wang C, Chen W, Zhang D, et al. Long Noncoding RNA LINC00941 Promotes Cell Proliferation and Invasion by Interacting with hnRNPK in Oral Squamous Cell Carcinoma. Nutrition and Cancer. 2022;74(8):2983-95.

14. Wang J, Meng W, Yang J, Cao H, Liu T, Yang C, et al. Aberrant methylation-mediated downregulation of the LINC01554 gene accelerates the malignant progression and regulates the chemosensitivity of laryngeal squamous cell carcinoma. J Physiol Pharmacol. 2022;73(2).

15. Wu J, Xie H. Expression of long noncoding RNA-HOX transcript antisense intergenic RNA in oral squamous cell carcinoma and effect on cell growth. Tumor Biology. 2015;36(11):8573-8.

16. Xu J, Bo Q, Zhang X, Lei D, Wang J, Pan X. lncRNA HOXA11-AS promotes proliferation and migration via sponging miR-155 in hypopharyngeal squamous cell carcinoma. Oncology Research. 2020;28(3):311-9.

17. Yang J, Shi X, Yang M, Luo J, Gao Q, Wang X, et al. Glycolysis reprogramming in cancer-associated fibroblasts promotes the growth of oral cancer through the lncRNA H19/miR-675-5p/PFKFB3 signaling pathway. International journal of oral science. 2021;13(1):12.

18. Zhou J, Li W, Jin T, Xiang X, Li M, Wang J, et al. Gene microarray analysis of lncRNA and mRNA expression profiles in patients with hypopharyngeal squamous cell carcinoma. International Journal of Clinical and Experimental Medicine. 2015;8(4):4862-82.

19. Jia HC, Wang X, Sun Z. Screening and validation of plasma long non-coding RNAs as biomarkers for the early diagnosis and staging of oral squamous cell carcinoma. ONCOLOGY LETTERS. 2021;21(2).

20. Lyu K, Li Y, Xu Y, Yue H, Wen Y, Liu T, et al. Using RNA sequencing to identify a putative lncRNA-associated ceRNA network in laryngeal squamous cell carcinoma. RNA Biology. 2020;17(7):977-89.

21. Liu L, Zhan Y, Huang Y, Huang L. LncRNA FGD5-AS1 can be predicted as therapeutic target in oral cancer. Journal of Oral Pathology and Medicine. 2020;49(3):243-52.

22. Zhang LM, Ju HY, Wu YT, Guo W, Mao L, Ma HL, et al. Long non-coding RNA ANRIL promotes tumorgenesis through regulation of FGFR1 expression by sponging miR-125a-3p in head and neck squamous cell carcinoma. American Journal of Cancer Research. 2018;8(11):2296-310.

23. Pan L, Chen H, Bai Y, Wang Q, Chen L. Long non-coding RNA CASC2 serves as a ceRNA of microRNA-21 to promote PDCD4 expression in oral squamous cell carcinoma. OncoTargets and Therapy. 2019;12:3377-85.

24. Sassenberg M, Droop J, Schulz WA, Dietrich D, Loick SM, Wiek C, et al. Upregulation of the long non-coding RNA CASC9 as a biomarker for squamous cell carcinoma. BMC Cancer. 2019;19(1).

25. Sun M, Shen Z. Knockdown of long non-coding RNA (lncRNA) colon cancer-associated transcript-1 (CCAT1) suppresses oral squamous cell carcinoma proliferation, invasion, and migration by inhibiting the discoidin domain receptor 2 (DDR2)/ERK/AKT axis. Medical Science Monitor. 2020;26.

26. Wang Q, Han J, Xu P, Jian X, Huang X, Liu D. Silencing of lncrna snhg16 downregulates cyclin d1 (Ccnd1) to abrogate malignant phenotypes in oral squamous cell carcinoma (oscc) through upregulating mir-17-5p. Cancer Management and Research. 2021;13:1831-41.

27. Wang Q, Zhang W. LncRNA PVT1 participates in the development of oral squamous cell carcinomas through accelerating EMT and serves as a diagnostic biomarker. Panminerva medica. 2021;63(3):396-7.

28. Kyurkchiyan SG, Popov TM, Stancheva G, Rangachev J, Mitev VI, Popova DP, et al. Novel insights into laryngeal squamous cell carcinoma from association study of aberrantly expressed miRNAs, lncRNAs and clinical features in Bulgarian patients. Journal of BUON. 2020;25(1):357-66.

29. Saproo S, Sarkar SS, Gautam V, Konyak CW, Dass G, Karmakar A, et al. Salivary protein kinase C alpha and novel microRNAs as diagnostic and therapeutic resistance markers for oral squamous cell carcinoma in Indian cohorts. Frontiers in Molecular Biosciences. 2023;9.

30. Zhang S, Wang X, Wang D. Long non-coding RNA LINC01296 promotes progression of oral squamous cell carcinoma through activating the MAPK/ERK signaling pathway via the miR-485-5p/PAK4 axis. Archives of Medical Science. 2022;18(3):786-99.

31. Zhou S, Zhu Y, Li Z, Zhu Y, He Z, Zhang C. Exosome-derived long non-coding RNA ADAMTS9-AS2 suppresses progression of oral submucous fibrosis via AKT signalling pathway. Journal of Cellular and Molecular Medicine. 2021;25(4):2262-73.

32. Kolenda T, Kapałczynska M, Teresiak A, Guglas K, Blizniak R, Łuczewski Ł, et al. LncRNAs as new biomarkers in head and neck cancers. Wspolczesna Onkologia. 2016;19:40.

33. Gao W, Zhang C, Ma T, Wen S, Fu R, Zhao D, et al. Potential biomarkers and their regulatory relationships in laryngeal squamous cell carcinoma with lymph node metastasis revealed by integrating mRNA, microRNA and long non-coding RNA profiles. International Journal of Clinical and Experimental Pathology. 2016;9(5):5103-16.

34. Qiu X, Lei Z, Wang Z, Xu Y, Liu C, Li P, et al. Knockdown of lncRNA RHPN1-AS1 inhibits cell migration, invasion and proliferation in head and neck squamous cell carcinoma. Journal of Cancer. 2019;10(17):4000-8.

35. Yang YF, Feng L, Shi Q, Ma HZ, He SZ, Hou LZ, et al. Silencing novel long non-coding RNA FKBP9P1 represses malignant progression and inhibits PI3K/AKT signaling of head and neck squamous cell carcinoma in vitro. Chinese medical journal. 2020;133(17):2037-43.

36. Yang YT, Wang YF, Lai JY, Shen SY, Wang F, Kong J, et al. Long non-coding RNA UCA1 contributes to the progression of oral squamous cell carcinoma by regulating the WNT/β-catenin signaling pathway. Cancer Science. 2016;107(11):1581-9.

37. Yang Y, Chen D, Liu H, Yang K. Increased expression of lncRNA CASC9 promotes tumor progression by suppressing autophagy-mediated cell apoptosis via the AKT/mTOR pathway in oral squamous cell carcinoma. Cell Death and Disease. 2019;10(2).

38. Zhao Y, Yao R. Long non-coding RNA HOXA-AS3 promotes cell proliferation of oral squamous cell carcinoma through sponging microRNA miR-218-5p. Bioengineered. 2021;12(1):8724-37.
